# Interferon-gamma Mediated Metabolic Pathways in Hospitalized Patients During Acute and Reconvalescent COVID-19

**DOI:** 10.1177/11786469231154244

**Published:** 2023-02-13

**Authors:** Mario Gietl, Francesco Burkert, Stefanie Seiwald, Anna Böhm, Stefanie Hofer, Johanna M Gostner, Talia Piater, Simon Geisler, Guenter Weiss, Judith Loeffler-Ragg, Thomas Sonnweber, Ivan Tancevski, Alex Pizzini, Sabina Sahanic, Dietmar Fuchs, Rosa Bellmann-Weiler, Katharina Kurz

**Affiliations:** Department of Internal Medicine II, Medical University Innsbruck, Biocentre, Medical Biochemistry, Innsbruck, Austria

**Keywords:** COVID-19, interferon-gamma, neopterin, tryptophan, phenylalanine, IDO, Long Covid

## Abstract

**Background:**

Fatigue, sleep disturbance, and neurological symptoms during and after COVID-19 are common and might be associated with inflammation-induced changes in tryptophan (Trp) and phenylalanine (Phe) metabolism.

**Aim:**

This pilot study investigated interferon gamma inducible biochemical pathways (namely Trp catabolism, neopterin, tyrosine [Tyr], and nitrite formation) during acute COVID-19 and reconvalescence.

**Patients and methods:**

Thirty one patients with moderate to severe COVID-19 admitted to the University Hospital of Innsbruck in early 2020 (March-May) were followed up. Neurotransmitter precursors Trp, Phe, Tyr as well as kynurenine (Kyn), neopterin, nitrite, and routine laboratory parameters were analyzed during acute infection and at a follow-up (FU) 60 days thereafter. Clinical symptoms of patients (neurological symptoms, fatigue, sleep disturbance) were recorded and associations with concentrations of laboratory parameters investigated.

**Results and conclusion:**

Almost half of the patients suffered from neurological symptoms (48.4%), the majority of patients experienced sleep difficulties (56.7%) during acute COVID-19. Fatigue was present in nearly all patients. C-reactive protein (CRP), interleukin-6 (IL-6), neopterin, Kyn, Phe concentrations were significantly increased, and Trp levels depleted during acute COVID-19. Patients with sleep impairment and neurological symptoms during acute illness presented with increased CRP and IL-6 concentrations, Trp levels were lower in patients with sleep disturbance. In general, inflammatory markers declined during reconvalescence. A high percentage of patients suffered from persistent symptoms at FU (neurological symptoms: 17.2%, fatigue: 51.7%, sleeping disturbance: 34.5%) and had higher CRP concentrations. Nitrite and Phe levels were lower in patients with sleeping difficulties at FU and Kyn/Trp ratio, as indicator of IDO activity, was significantly lower in patients with neurological symptoms compared to patients without them at FU. In summary, inflammation induced alterations of amino acid metabolism might be related to acute and persisting symptoms of COVID-19.

## Introduction

Severe acute respiratory syndrome coronavirus (SARS-CoV-2) was first registered in 2019 in Wuhan City, China. The SARS-CoV-2 virus causes the coronavirus disease 2019 (COVID-19), which mostly presents as a mild asymptomatic infection, but may also cause a severe life-threatening disease. The course of the disease depends on the quantity and pathogenicity of the invading specific virus variants,^1-3^ as well as on the integrity of the mucosal barriers^4^ and the efficacy of the immune system against the viral infection. Co-morbidities, environmental and genetic factors shape the individual immune status.^5,6^ Moreover, dysfunctional systemic immune response (cytokine release syndrome) is associated with severe disease and a worse outcome for patients.^5,7-9^

The pro-inflammatory cytokines interleukin 6 (IL-6) and interferon-gamma (IFN-γ) play a key role in the orchestration of inflammatory cascades and are strongly elevated in patients with acute COVID-19.^10-13^ Recent studies have shown that COVID-19 infection induces IFN-γ mediated Trp catabolism and neopterin formation, which both predict a poor outcome.^9,14,15^ Interestingly, also Trp metabolites Kyn, 3-hydroxykynurenine, and 3-hydroxyanthranilate (measured in the urine of patients with critical and non-critical COVID-19 infection) were shown recently to be associated with disease severity and systemic inflammation.^16^ Urine metabolome analyses also showed that aminoaciduria is common during SARS-CoV-2 infection whereby higher levels of Phe, proline and leucine, and Tyr were excreted in non-critical and critical COVID-19 patients compared to healthy controls with a tendency for the greatest increase among critical patients.^16^

However, activation of IFN-γ mediated pathways is not only predictive for the outcome of patients, but may also contribute importantly to the development of symptoms during acute COVID-19, and also persisting symptoms after infection (Long Covid).^17^ Coronavirus infections have been associated with long-term complications after recovery also in the pre-COVID-19 era, however the enormous number of individuals experiencing SARS-CoV-2 and the high prevalence of persisting symptoms (median frequency of 72.5%, assessing 16 studies reporting at least one symptom being present at follow-up) lead to a great individual burden with consequences for health care and economic systems.^18,19^

**Figure 1.**
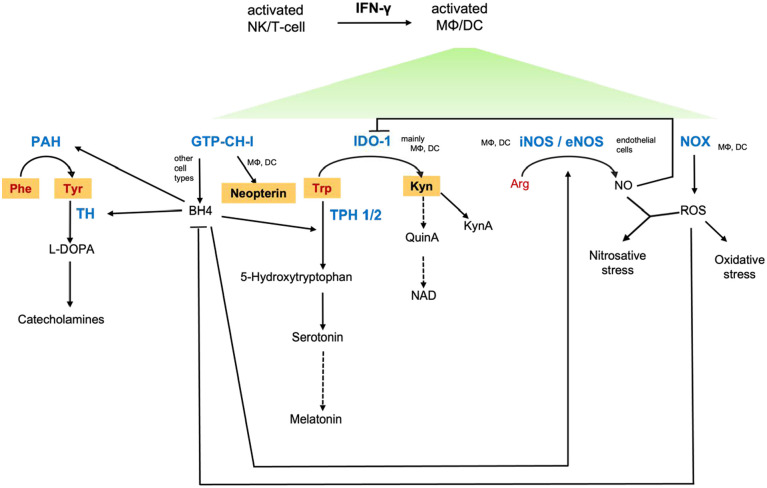
Interferon gamma (IFN-γ)-dependent and related biochemical pathways. Inflammatory cascades stimulate immunobiochemical pathways, with IFN-γ being the main activating cytokine of neopterin formation via GTP cyclohydrolase 1 (GTP-CH-I) and tryptophan (Trp) catabolism along the kynurenine (Kyn) axis via indoleamine 2,3-dioxygenase 1 (IDO-1) in human macrophages (MΦ) and dendritic cells (DC). The shift toward neopterin production runs at the expense of tetrahydrobiopterin (BH4) in these cell types. BH4 is crucial for the functioning of several monoxygenases, for example phenylalanine 4-monooxygenase (PAH), tyrosine 3-monooxygenase (TH), tryptophan 5-monooxygenases (TPH), and nitric oxide synthases (NOS). Though BH4 is synthetized by other cell types, too, it is oxidation labile and availabilities may become limiting. In addition, a prooxidative milieu leads to dysbalances in the Kyn downstream axis, leading to immunological and neurological consequences. Abbreviations: KynA, kynurenic acid; NAD, nicotinamide adenine dinucleotide; NK, neutral killer cells; NO, nitric oxide; NOX, NADPH oxidase; Phe, phenylalanine; Arg, arginine; QuinA, quinolinic acid; ROS, reactive oxygen species; Tyr, tyrosine. Metabolites analyzed in this study are in bold and highlighted in orange. Enzymes are shown in blue and amino acids in red text.

Earlier studies in patients with human immunodeficiency virus (HIV) infection and in cancer patients have shown that activation of IFN-γ mediated pathways is related to fatigue, depression, immunodeficiency, and an impaired quality of life.^20-24^ IFN-γ has a central function in the metabolic reprogramming of immune cells and also other responsive cells, causing far-reaching signal changes that have effects far beyond immunological processes. Thus, IFN-γ signaling may affect various biochemical pathways important for neurotransmitter synthesis, sleep, immune response, and circulation (Figure 1). COVID-19 infection goes along with fatigue, sleep disturbances, mood alterations, endothelial dysfunction, and neurological symptoms. Therefore, we hypothesize that immune-mediated metabolic changes might be responsible for the development of these symptoms in patients.

Among the IFN-γ dependent pathways, induction of the enzyme indoleamine 2,3-dioxygenase 1 (IDO1)^23,25^ appears to be of utmost importance: Enhanced catabolism of Trp to Kyn inhibits the proliferation of pathogens,^26^ but also of T-cells, thus crucially influencing the efficacy of immune cascades.^27,28^ Furthermore, enhanced Trp catabolism results in the accumulation of neurotoxic catabolites (like quinolinic acid, kynurenic acid, or 3-hydroxy-kynurenine^29,30^) and might impair serotonin and melatonin formation. Thus, decreased Trp availability might go along with increased anxiety, cognition deficits, or depressed mood and sleep disturbances due to serotonin deficits.^31-34^ In fact, reduced levels of serotonin and melatonin have also been associated with COVID-19 severity and death recently in a study using untargeted metabolomics of COVID-19 sera.^35^

Similarly, decreased availability of the pivotal enzymatic cofactor 5,6,7,8-tetrahydrobiopterin (BH4) impairs the synthesis of catecholamines as this cofactor is required by both phenylalanine 4-monoxygenase and tyrosine 3-monooxygenase. Moreover, low BH4 impairs the formation of the vasodilating molecule nitric oxide (NO) and the serotonin precursor 5-hydroxytryptophan (see Figure 1): BH4 can be oxidized quickly by reactive oxygen species (ROS), which are formed in parallel with neopterin during immune response.^36^ Increased ROS production depletes antioxidants leading to oxidative stress. Neopterin is a reliable biomarker for the extent of oxidative stress and for monitoring Th1 type immune activation.^37^ As ROS can furthermore react with NO to form peroxynitrite and nitrotyrosine, nitrosative stress can develop with impairment of endothelial relaxation and immune cascades.^38^ Whether increased Kyn formation may also be a feedback mechanism due to its potential vasorelaxing activity still needs to be discussed including its role in inflammation induced hypotension.^39,40^

IFN-γ signaling thus can have significant effects on immune response,^41^ neurotransmitter synthesis^29^ and circulation^42^ in patients with acute COVID-19: Impairment of Trp and Phe metabolism^29,43^ and decreased NO availability^44^ have been demonstrated in COVID-19 infected patients and might in fact impair their physical and mental performance. These metabolic alterations could thus contribute importantly to the development of neurological symptoms like headache, dizziness, and generalized weakness, which have been frequently observed in COVID-19 patients.^45-47^ In our pilot study we investigated whether neurological symptoms, sleep disturbance, and fatigue were related to metabolic changes induced by inflammation and IFN-γ-mediated pathways in patients with acute COVID-19 and at FU.

## Materials and Methods

### Participants and study design

**Figure 2.**
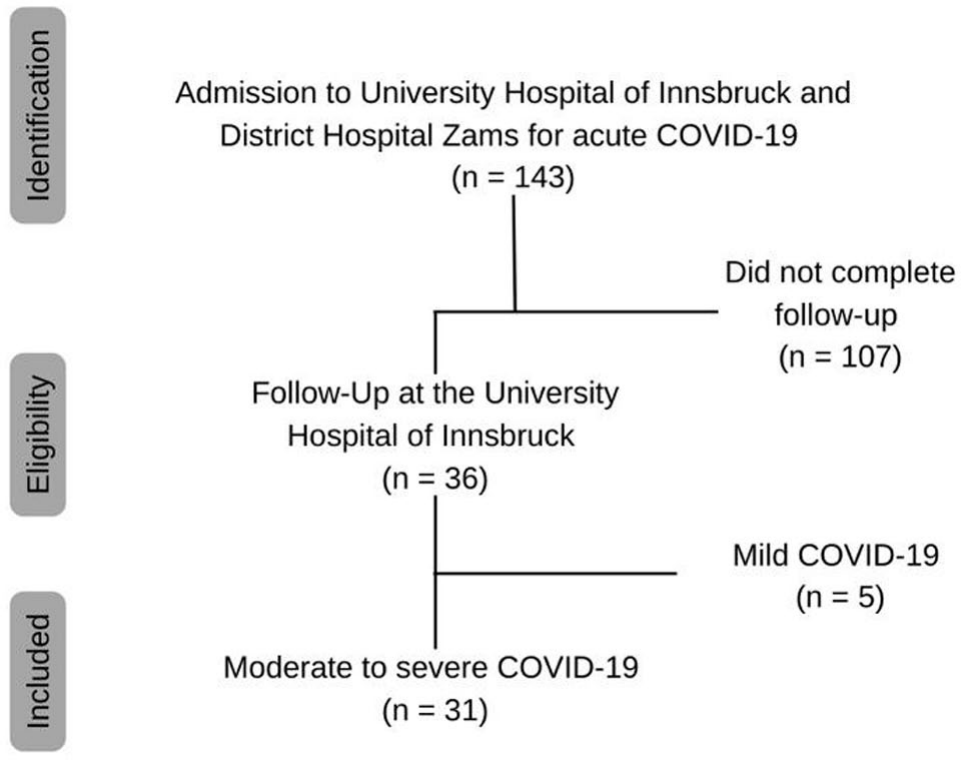
Flow chart.

Of 143 patients, who were admitted to the University Hospital of Innsbruck and the District Hospital Zams for treatment of moderate to severe COVID-19 between March and May 2020, 31 patients came to the outpatient department for a follow-up investigation 60 days after symptom onset^48^ (Figure 2). The number of patients coming for FU was rather low, as many patients who were admitted to the hospitals in Innsbruck or Zams were originally from other countries and returned home afterward, while others were still on rehabilitation. Furthermore, we only could analyze blood samples from patients who were treated initially in Innsbruck and had a moderate to severe course of disease. The follow-up (FU) included a clinical evaluation, blood sampling and a questionnaire. Epidemiological data and patients’ symptoms/medical history were recorded and blood samples were analyzed as described below. The patients gave informed consent for their data to be used for scientific research and the studies were approved by the Ethical Board of the Medical University of Innsbruck (1167/2020 and 1157/2017).

### Laboratory analysis

Routine laboratory parameters were analyzed by the Central Institute for Medical and Chemical Laboratory Analysis in Innsbruck. IFN-γ dependent parameters were analyzed at the Institute of Medical Biochemistry, Biocenter, Medical University of Innsbruck. Aromatic amino acids and the Trp catabolite Kyn were determined by HPLC using a method based on the protocols of Widner et al^49^ and Neurauter et al.^50^ Briefly, chromatographic separation was performed on an Agilent 1260 HPLC system with a LiChroCART 55-4 C18 column (3 µm particle size, Merck, Darmstadt, Germany) connected to a C-18 security guard precolumn (4 mm × 3.0 mm, Phenomenex, California, USA), using 15 mM potassium dihydrogen phosphate buffer as mobile phase, isocratic elution at a flow rate of 1.1 mL/min at 25°C. Internal as well as calibration standards (Sigma Aldrich, Austria) were dissolved in aqueous albumin solution (70 g/L, AL-Labortechnik, Zeillern-Amstetten, Austria). In each case, 100 µL of the internal standard solution (3-nitro-l-tyrosine, 25 µM) was added to 100 µL of the samples or to the calibration standards. Proteins were precipitated with 25 µL trichloroacetic acid (2M) and precipitates were removed by centrifugation. Kyn and the internal standard 3-nitro-l-tyrosine were determined at a wavelength of 360 nm using a 1260 Infinity II DAD detector (G7115A). Trp, Phe, and Tyr were determined via their native fluorescence using a 1260 Infinity II fluorescence detector (G7121B) (Trp: excitation wavelength of 286 nm, emission wavelength of 366 nm; Phe and Tyr: excitation wavelength of 210 nm, emission wavelength of 302 nm).

Neopterin concentrations were measured by ELISA (BRAHMS Diagnostica, Berlin, Germany), and nitrite was determined by a modified Griess reaction.^51^ The ratios of kynurenine to tryptophan (Kyn/Trp) and phenylalanine to tyrosine (Phe/Tyr) were calculated as surrogate for IDO-1^51^ and PAH-activity.^52^

### Questionnaire

Sleep impairment and current medication were included in the questionnaire, which was given to the patients 60 days after symptom onset (FU). Accordingly, clinical symptoms during acute COVID-19 were assessed retrospectively at FU. Sleep disturbance was noted to be present if mentioned by the patient or prescription of sleep medication. Furthermore, the Eastern Cooperative Oncology Group (ECOG) score was part of the questionnaire.^53^

### Additional data and definitions

The presence of neurological symptoms was recorded from the patients’ medical records: a broad spectrum of neurological manifestations was reported. Data on fatigue were obtained through the structured medical interview (in line with these records all patients reporting fatigue had an ECOG score ⩾2).

### Statistical analysis

Categorical variables were displayed as frequencies and percentages, and non-normally distributed continuous variables were assessed using median, interquartile range, and/or range. The majority of variables were not normally distributed, thus non-parametric tests were applied: Mann-Whitney U-test was used to compare 2 independent groups, and the Wilcoxon test yielded results for 2 dependent groups. Correlations were evaluated using the Spearman rank test. Alpha was prespecified at .05 and was used for all statistical tests. All analyses were performed using IBM SPSS Statistics 28.0 (IBM Corp., USA).

## Results

### Baseline characteristics

Thirty-one patients were included in the final analysis, the majority of the study participants (N = 22, 71%) were male. The median age was 61 years (interquartile range (IQR) 54-71 years) ranging from 44 to 87 years.

### Clinical symptoms

In Table 1 occurrence of clinical symptoms during acute COVID-19 and 60 days after the onset of symptoms is shown. Nearly all patients suffered from fatigue (n = 30) and more than half of our study population reported sleep disturbances during acute infection (n = 17). Neurological symptoms were prevalent in almost half of the patients (n = 15). Over time all symptoms regressed.

**Table 1.** Frequencies of nominal study parameters during acute COVID-19 and at follow-up (FU, n = 31).

	Neurological symptoms		Fatigue		Sleep disturbance	
	n (valid %)	Missing	n (valid %)	Missing	n (valid %)	Missing
Acute	15 (48.4)	0	30 (100)	1	17 (56.7)	1
FU	5 (17.2)	2	15 (51.7)	2	10 (34.5)	2

n, number of patients; missing, number of patients with missing value; valid %, percentage of patients with a valid value.

### Laboratory parameters

Numerous laboratory parameters showed significant differences between acute illness and the 60 days follow-up as depicted in Table 2. Apart from Tyr (64.99 vs 58.34 µmol/L, *P* = .063), all IFN-γ related parameters differed significantly between acute COVID-19 and FU. Neopterin (29.17 vs 9.09 nmol/L, *P* < .001), Kyn (3.42 vs 2.32 µmol/L, *P* = .004), and Phe (126.57 vs 68.91 µmol/L, *P* < .001) were higher, and Trp concentrations (43.29 vs 52.80 µmol/L, *P* = .024) lower during acute COVID-19. Regarding inflammatory parameters, CRP (10.93 vs 0.16 mg/dL, *P* < .001) and IL-6 (85.8 vs 2.75 ng/L, *P* < .001) were significantly higher in patients with an acute infection. Iron metabolism markers like total serum iron (4.35 vs 15.7 µmol/L, *P* < .001), transferrin (141.5 vs 234 mg/dL, *P* < .001), and transferrin saturation (12.5% vs 25.5%, *P* = .001) were depleted during active illness.

**Table 2.** Study parameters and comparison between parameters during acute COVID-19 (acute) and follow-up (FU).

	Acute	FU	*P*-value*
	Median (IQR)	Median (IQR)	
**Neopterin [nmol/L]**	**29.17 (17.89-44.75)**	**9.09 (8.0-13.35)**	<**.001**
**Kyn [µmol/L]**	**3.42 (2.35-4.05)**	**2.32 (1.94-2.49)**	**.004**
**Trp [µmol/L]**	**43.29 (37.74-52.17)**	**52.80 (46.49-60.55)**	**.024**
**Phe [µmol/L]**	**126.57 (113.24-150.35)**	**68.91 (62.59-80.42)**	<**.001**
Tyr [µmol/L]	64.99 (59.16-74.54)	58.34 (53.06-72.88)	.063
**Kyn/Trp [µmol/mmol]**	**67.36 (53.63-92.64)**	**41.01 (34.42-56.79)**	<**.001**
**Phe/Tyr [µmol/µmol]**	**1.91 (1.59-2.15)**	**1.11 (0.99-1.36)**	<**.001**
Nitrite [µmol/L]	22.66 (12.85-41.14)	29.62 (12.69-96.96)	n.s.
WBC [G/L]	6.3 (4.9-8.6)	6.6 (5.38-7.43)	n.s.
**Segmented neutrophils [%]**	**78.0 (60.0-85.5)**	**60.65 (53.33-70.28)**	**.002**
Monocytes [%]	5.1 (2.8-9.4)	8.1 (6.23-9.33)	n.s.
**Eosinophil granulocytes [%]**	**0.2 (0.0-1.4)**	**1.5 (0.93-2.58)**	<**.001**
**Lymphocytes [% of WBC]**	**15 (9.4-26.3)**	**26 (21.08-32.58)**	**.001**
**Lymphocytes [G/L]**	**0.95 (0.58-1.25)**	**1.58 (1.23-2.14)**	<**.001**
**Fibrinogen [mg/dL]**	**582 (450-637.5)**	**300 (256-387.8)**	<**.001**
**CRP [mg/dL]**	**10.93 (2.72-19.04)**	**0.16 (0.06-0.61)**	<**.001**
**Procalcitonin [µg/L]**	**0.2 (0.08-0.5)**	**0.06 (0.06-0.07)**	<**.001**
**IL-6 [ng/L]**	**85.8 (21.2-205.4)**	**2.75 (1.5-7.43)**	<**.001**
Hemoglobin [g/L]	123 (116-137)	132.5 (122-140.75)	n.s.
RBC [T/L]	3.96 (3.71-4.48)	4.24 (3.96-4.6)	n.s.
**MCV [fL]**	**89 (86.6-92.4)**	**91.4 (89.78-94.18)**	<**.001**
MCH [pg]	30.9 (30.2-31.6)	30.45 (29.8-31.63)	n.s.
Folate [µg/L]	10.6 (5.43-13.25)	6.5 (4.43-9.6)	n.s.
**Vitamin B12 [pmol/mL]**	**396.5 (291.25-654)**	**285 (207.75-372.5)**	**.040**
**Total serum iron [µmol/L]**	**4.35 (3.23-5.7)**	**15.7 (9.08-19.95)**	<**.001**
**Ferritin [µg/L]**	**973 (494-2021)**	**253 (135-413.75)**	<**.001**
**Transferrin [mg/dL]**	**141.5 (108.75-177.25)**	**234 (210.75-264)**	<**.001**
**Transferrin saturation [%]**	**12.5 (10-17.75)**	**25.5 (13.75-35.5)**	**.001**

Median values and IQR of study parameters and longitudinal comparison between certain groups.

* *P*-value comparing parameters during acute COVID-19 (acute) and follow-up (FU); no significant difference (n.s.). Lab parameters printed in bold differed significantly between acute Covid -19 infection and reconvalescence.

### Comparison of study parameters during acute COVID-19

**Figure 3.**
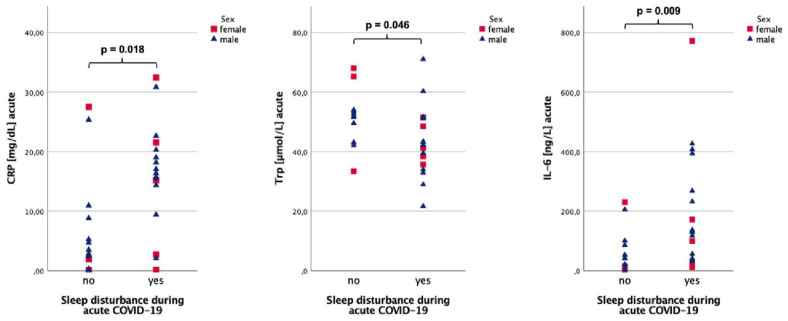
CRP, IL-6, and Trp serum concentrations during acute COVID-19 sex-stratified for sleep disturbance during acute COVID-19.

During acute COVID-19, CRP (16.37 vs 3.5 mg/dL, *P* = .018) and IL-6 concentrations (130.8 vs 41.6 ng/L, *P* = .009) were significantly higher and Trp levels (42.2 vs 51.8 µmol/L, *P* = .046) lower in patients with sleeping difficulties (Figure 3). Higher CRP was recorded when neurological symptoms were present (16.37 vs 3.46 mg/dL, *P* = .053). For detailed results see Table 3.

**Table 3.** Comparison of neurological symptoms and sleep disturbance during acute COVID-19.

	Neurological symptoms		*P*-value*	Sleep disturbance		*P*-value**
	No	Yes		No	Yes	
	(n = 16)	(n = 15)		(n = 13)	(n = 17)	
	Median (IQR)	Median (IQR)		Median (IQR)	Median (IQR)	
Neopterin [nmol/L]	42.5 (16.0-47.6)	24.9 (18.3-36.6)	n.s.	32.6 (17.1-43.6)	24.9 (17.3-45.1)	n.s.
Kyn [µmol/L]	3.4 (2.4-3.9)	3.4 (1.9-4.3)	n.s.	3.7 (2.5-4.1)	3.1 (2.1-3.9)	n.s.
Trp [µmol/L]	43.1 (38.4-51.9)	51.3 (35.7-53.6)	n.s.	**51.8 (43.1-53.9)**	**42.2 (34.8-51.4)**	**.046**
Phe [µmol/L]	131.6 (121.4-151.3)	120.5 (112.0-130.0)	n.s.	128.8 (117.9-132.0)	122.6 (98.2-153.6)	n.s.
Tyr [µmol/L]	62.6 (60.2-77.9)	70.9 (57.9-73.4)	n.s.	68.2 (58.7-73.3)	63.5 (58.0-81.3)	n.s.
Kyn/Trp [µmol/mmol]	68.1 (57.3-96.7)	66.7 (47.5-89.6)	n.s.	69.1 (48.7-87.2)	63.2 (52.6-94.1)	n.s.
Phe/Tyr [µmol/µmol]	2.0 (1.55-2.2)	1.8 (1.6-2.0)	n.s.	2.0 (1.8-2.1)	1.7 (1.5-2.2)	n.s.
Nitrite [µmol/L]	22.9 (11.6-33.7)	22.4 (13.3-44.0)	n.s.	28.3 (13.4-43.7)	20.4 (9.3-34.5)	n.s.
CRP [mg/dL]	3.46 (2.18-14.17)	16.37 (10.93-21.53)	0.053	**3.5 (1.1-9.9)**	**16.37 (11.88-20.92)**	**.018**
IL-6 [ng/L]	41.5 (14.2-135.0)	118.1 (42.0-230.2)	n.s.	**41.6 (9.9-93.1)**	**130.8 (48.7-331.2)**	**.009**

Median values and IQR of study parameters during acute COVID-19.

* *P*-value comparing the 2 groups of the categorical variable neurological symptoms.

** *P*-value comparing the 2 groups of the categorical variable sleep disturbance; no significant difference (n.s.). Lab parameters printed in bold differed significantly between patients with/without sleep disturbance.

When studying for sex specific differences, men with sleeping problems had significantly higher CRP (16.72 vs 4.10 mg/dL, *P* = .007) and IL-6 levels (133.8 vs 41.8 ng/L, *P* = .007) compared to men without them, while women with sleep disturbances had no differences in CRP or IL-6 levels, but higher Kyn/Trp (91.3 vs 44.7, *P* = .025) than women with undisturbed sleep. Additionally, CRP was higher in men, when neurological symptoms were present (16.72 vs 4.38 mg/dL, *P* = .018).

**Figure 4.**
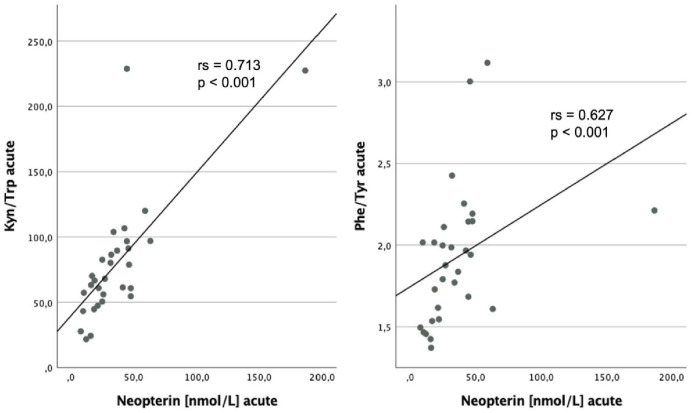
Relationship between neopterin levels during acute COVID-19 and Kyn/Trp and Phe/Tyr during acute illness respectively, with corresponding results yielded by Spearman Rank Test.

Neopterin levels were markedly associated with Kyn (rs = 0.646; *P* < .001), Trp (rs = −0.415; *P* = .023), Kyn/Trp (rs = 0.713; *P* < .001), Phe (rs = 0.702; *P* < .001), and Phe/Tyr (rs = 0.627; *P* < .001) during acute illness (Figure 4). Patients with high Phe levels also had elevated Kyn/Trp (rs = 0.426; *P* = .019) and high nitrite concentrations (rs = 0.371; *P* = .043).

### Comparison of concentrations of study parameters at follow-up (FU)

**Figure 5.**
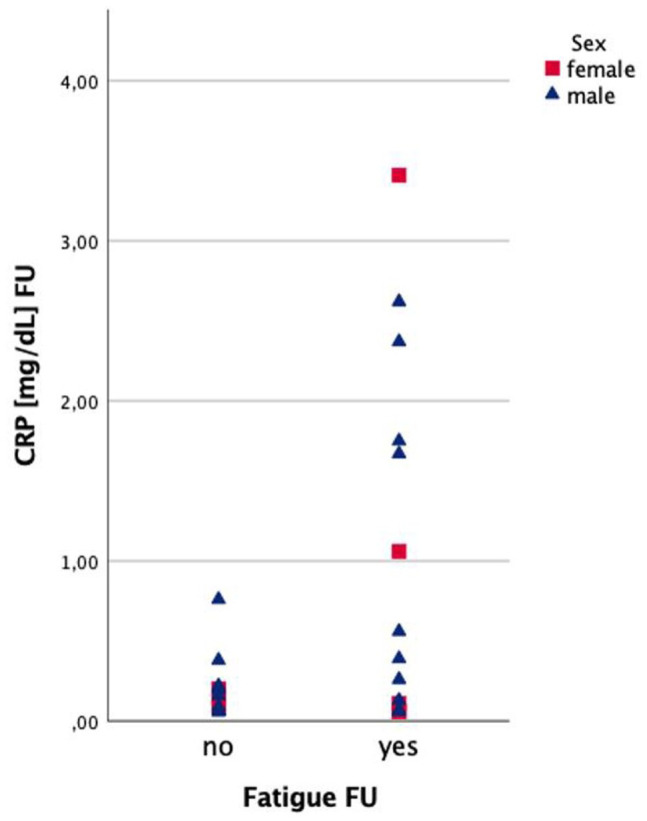
CRP concentrations 60 days after acute COVID-19 (FU) sex-stratified for fatigue.

**Figure 6.**
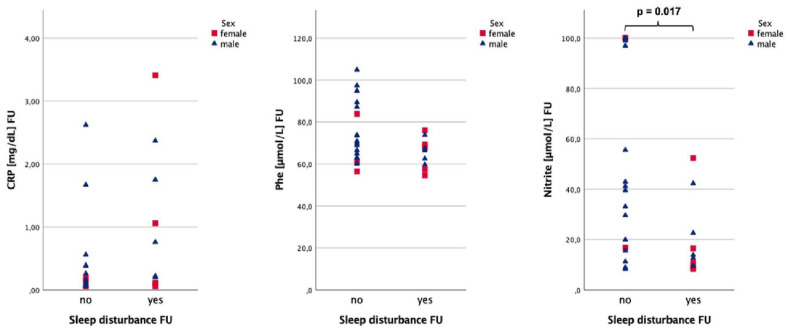
Nitrite, CRP, and Phe concentrations at FU sex-stratified for sleep disturbance at FU.

**Figure 7.**
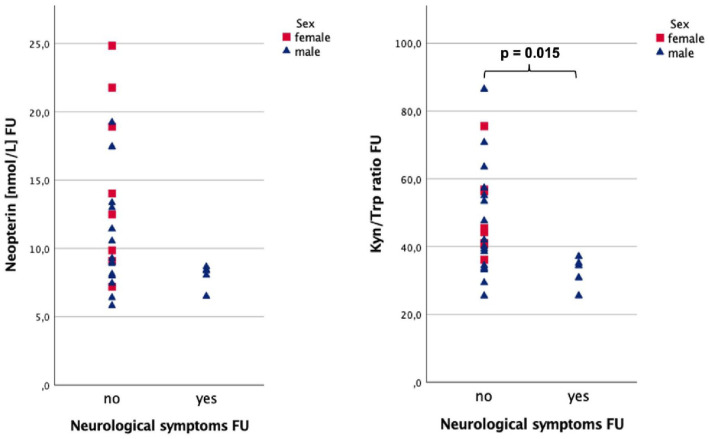
Neopterin and Kyn/Trp at FU respectively sex-stratified for neurological symptoms at FU.

Patients with fatigue tended to have higher serum CRP levels (0.39 vs 0.12 mg/dL, *P* = .055) 60 days after the onset of symptoms (Figure 5). Patients reporting sleep disturbance (see Figure 6) at follow-up had significantly lower nitrite concentrations (13.3 vs 41.2 µmol/L, *P* = .017) and tended to have higher CRP (0.49 vs 0.13 mg/dL, *P* = .060) at FU. Additionally, a trend was observed for lower Phe levels (67.0 vs 70.9 µmol/L, *P* = .081) in the same group. When neurological symptoms were still present (see Figure 7) Kyn/Trp was significantly lower (34.4 vs 43.1, *P* = .015), while neopterin tended to be lower (8.4 vs 10.2 nmol/L, *P* = .073). The full results are depicted in Table 4.

**Table 4.** Comparison of neurological symptoms, fatigue, and sleep disturbance at the follow-up (FU).

	Neurological symptoms		*P*-value*	Fatigue		*P*-value**	Sleep disturbance		*P*-value***
	No	Yes		No	Yes		No	Yes	
	(n = 24)	(n = 5)		(n = 14)	(n = 15)		(n = 19)	(n = 10)	
	Median (IQR)	Median (IQR)		Median (IQR)	Median (IQR)		Median (IQR)	Median (IQR)	
Neopterin [nmol/L]	10.2 (8.0-16.6)	8.4 (7.3-8.6)	.073	9.0 (7.9-13.5)	9.3 (8.0-17.4)	n.s.	8.9 (8.0-14.0)	10.2 (8.3-14.1)	n.s.
Kyn [µmol/L]	2.3 (2.0-2.8)	1.8 (1.6-2.6)	n.s.	2.4 (1.9-3.2)	2.2 (1.9-2.4)	n.s.	2.3 (2.0-3.0)	2.3 (1.9-2.5)	n.s.
Trp [µmol/L]	53.6 (47.4-58.9)	63.1 (50.3-75.7)	n.s.	54.5 (46.1-60.0)	52.8 (51.7-63.1)	n.s.	54.4 (51.9-63.1)	52.5 (42.8-57.5)	n.s.
Phe [µmol/L]	68.4 (61.4-81.9)	69.9 (64.8-84.4)	n.s.	70.1 (62.2-89.1)	67.9 (62.7-73.9)	n.s.	70.9 (63.1-89.4)	67.0 (59.2-70.5)	.081
Tyr [µmol/L]	59.0 (52.9-74.1)	67.2 (55.7-98.1)	n.s.	64.3 (52.5-76.3)	58.3 (54.2-70.5)	n.s.	58.3 (54.0-74.5)	59.7 (52.7-69.5)	n.s.
Kyn/Trp [µmol/mmol]	**43.1 (36.8-56.7)**	**34.4 (28.2-36.1)**	**.015**	44.8 (35.4-58.9)	39.3 (34.4-45.5)	n.s.	40.0 (33.7-55.1)	40.9 (36.4-56.5)	n.s.
Phe/Tyr [µmol/µmol]	1.1 (1.0-1.4)	1.0 (0.9-1.2)	n.s.	1.1 (1.0-1.4)	1.0 (0.96-1.3)	n.s.	1.1 (1.0-1.4)	1.0 (0.9-1.2)	n.s.
Nitrite [µmol/L]	24.8 (11.1-54.8)	42.9 (16.9-100)	n.s.	34.6 (11.9-97.9)	22.6 (11.2-52.4)	n.s.	**41.2 (16.8-100)**	**13.3 (9.3-27.5)**	**.017**
CRP [mg/dL]	0.16 (0.06-0.52)	0.26 (0.1-2.5)	n.s.	0.12 (0.06-0.21)	0.39 (0.07-1.75)	0.055	0.13 (0.06-0.38)	0.49 (0.11-1.91)	.060
IL-6 [ng/L]	2.5 (1.5-5.2)	8.5 (1.5-23.2)	n.s.	1.7 (1.5-4.9)	4.8 (1.5-12.7)	n.s.	1.8 (1.5-5.1)	5.3 (1.5-13.5)	n.s.

Median values and IQR of study parameters at FU.

* *P*-value comparing the 2 groups of the categorical variable neurological symptoms at FU.

** *P*-value comparing the 2 groups of the categorical variable fatigue at FU.

*** *P*-value comparing the 2 groups of the categorical variable sleep disturbance at FU; no significant difference (n.s.). Lab parameters printed in bold differed significantly between patients with/without neurological symptoms.

CRP was higher in men with sleeping problems (0.76 vs 0.11 mg/dL, *P* = .045) and fatigue (0.48 vs 0.09 mg/dL, *P* = .025) respectively compared to men without such symptoms.

## Discussion

Our data shows strong induction of inflammation and IFN-γ-dependent biochemical pathways in patients with acute and post-acute COVID-19. Trp catabolism and changes in Phe and Tyr metabolism were strongly induced during acute illness, while inflammatory and immune responses declined significantly after COVID-19. However, even 60 days after infection Trp catabolism and Phe accumulation were not normalized.

IDO-1 activity (as reflected by increased Kyn/Trp ratio), was markedly elevated during acute infection, which is well in line with earlier studies in patients with COVID-19,^29^ but also other infectious diseases (HIV, Influenza, Dengue virus, EBV)^21,54-56^ and cancer.^23^ Increased Trp catabolism went along with enhanced neopterin formation and Phe accumulation (as reflected by Phe/Tyr), which fits well with past findings in diabetes, Dengue virus, and cancer.^23,54,57^

Associations between inflammatory markers and amino acids indicate that pro-inflammatory cytokines induce biochemical pathways during acute COVID-19 and reconvalescence and that immune-mediated changes of amino acid metabolism might also be related to the development of symptoms: Neurological symptoms were present in almost half of the patients during infection. This finding suggests a strong impairment of neurological processes in hospitalized patients due to COVID-19, which builds on existing evidence.^58-60^ Sleep disturbances have been associated previously with increased levels of inflammatory parameters IL-6 and CRP,^61^ also in our population of COVID-19 patients higher IL-6 and CRP concentrations in patients were related with neurological symptoms and sleep disturbance during acute infection. Lower Trp concentrations in patients with sleep disturbances on the other hand might indicate that serotonin and melatonin formation were affected, however, we did not measure these parameters.

In the reconvalescent phase, that is 2 months after COVID-19, clinical symptoms like sleep disturbances, neurological symptoms, and fatigue had regressed in many patients, however, they were still present in a significant percentage of individuals (neurological symptoms: 17.2%, fatigue: 51.7%, sleeping disturbance: 34.5%). Still, symptoms were related to the extent of inflammation: Kyn/Trp was lower in patients with neurological symptoms at FU, and CRP levels were also significantly higher in male patients with fatigue and sleep disturbance at FU.

Patients with sleep problems also had tendentially lower Phe and significantly lower nitrite levels, which has not been described in patients with COVID-19 before. However, interestingly, a recent study in patients with chronic obstructive pulmonary disease showed that treatment with beetroot juice (which contains nitrates that are converted to nitrites and NO) was helpful to improve overnight sleep quality and the duration of sleep episodes—however without affecting serum nitrite concentrations in the morning.^62^

Limitations of this pilot study include the rather small sample size (with lacking statistical corrections for multiple comparisons) and the fact that we only had blood samples from 2 time points. Additionally, a selection bias in relation to those who turned up for the follow-up visit might have played a role, especially young people with few to no risk factors have often not been admitted to a hospital during severe acute COVID-19 but can also suffer from on-going complaints. For future research in this area, it would also be advisable to measure clinical symptoms more objectively with validated scales instead of relying on patient reports.

On the other hand, blood samples of patients were taken in the first wave of the pandemic, where blood sampling was not based on hypotheses about disease progression, and knowledge about the pathophysiology of COVID-19 was still largely unclear. COVID-19 patients were not treated with corticosteroids at that time; thus, our results are not biased by anti-inflammatory treatment—which might mask the impact of inflammatory pathways on clinical symptoms of patients.

To conclude, our pilot study could show that IFN-γ mediated biochemical pathways are induced during acute COVID-19 infection and reconvalescence and are related to symptoms of patients.

The associations between symptoms and the investigated inflammation markers in our pilot study indicate that the pathomechanisms that were in focus are relevant, though a causal cannot be proofed so far. To further support our hypothesis, it is required to monitor additional down-stream metabolites (eg, neurotoxic metabolites like quinolinic acid or 3-hydroxykynurenine) during infection and reconvalescence in larger cohorts of patients and prospectively. We postulate that the investigated parameters may contribute to a metaboinflammatory signature employable to assess the severity and course of disease and predict unfavorable outcomes. Nevertheless, our study is limited due to the low number of patients, thus further studies with larger cohorts are needed to confirm the validity of these signatures and also the dynamics of the biochemical pathways during acute infection and reconvalescence.

Our results strongly support the hypothesis that immune-mediated alterations of amino acid metabolism may provide a biochemical rationale why and how patients develop certain symptoms. We propose that studies using larger metabolomic approaches, which cover a broader range of pathways such as formation of downstream metabolites like serotonin, melatonin, quinolinic acid, NAD, dopamine, and catecholamines, etc. should be performed to comprehensively assess the metabolic shift that is associated with post-infectious symptoms, and finally to translate these findings in personalized treatment options.
